# The Role of Salicylic Acid in Plants Exposed to Heavy Metals

**DOI:** 10.3390/molecules25030540

**Published:** 2020-01-26

**Authors:** Anket Sharma, Gagan Preet Singh Sidhu, Fabrizio Araniti, Aditi Shreeya Bali, Babar Shahzad, Durgesh Kumar Tripathi, Marian Brestic, Milan Skalicky, Marco Landi

**Affiliations:** 1 State Key Laboratory of Subtropical Silviculture, Zhejiang A&F University, Hangzhou 311300, China; 2 Department of Environment Education, Government College of Commerce and Business Administration, Chandigarh 160047, India; gagan1986sidhu@gmail.com; 3 Dipartimento AGRARIA, Università Mediterranea di Reggio Calabria, Località Feo di Vito, SNC I-89124 Reggio Calabria, RC, Italy; 4 Mehr Chand Mahajan D.A.V. College for Women, Chandigarh 160036, India; shreeyaaditi02@gmail.com; 5 School of Land and Food, University of Tasmania, Hobart, TAS 7005, Australia; babar.shahzad@utas.edu.au; 6 Amity Institute of Organic Agriculture, Amity University Uttar Pradesh, Noida 201313, India; dktripathiau@gmail.com; 7 Department of Plant Physiology, Faculty of Agrobiology and Food Resources, Slovak University of Agriculture, 94976 Nitra, Slovakia; marian.brestic@uniag.sk; 8 Department of Botany and Plant Physiology, Faculty of Agrobiology, Food and Natural Resources, Czech University of Life Sciences, 16500 Prague, Czech Republic; skalicky@af.czu.cz; 9 Department of Agriculture, Food and Environment, University of Pisa, I-56124 Pisa, Italy; 10 CIRSEC, Centre for Climatic Change Impact, University of Pisa, Via del Borghetto 80, I-56124 Pisa, Italy; 11 Interdepartmental Research Center Nutrafood “Nutraceuticals and Food for Health”, University of Pisa, I-56124 Pisa, Italy

**Keywords:** metal toxicity, *ortho*-hydroxybenzoic acid, plant hormone, metal pollution, polyphenols, signaling compound

## Abstract

Salicylic acid (SA) is a very simple phenolic compound (a C_7_H_6_O_3_ compound composed of an aromatic ring, one carboxylic and a hydroxyl group) and this simplicity contrasts with its high versatility and the involvement of SA in several plant processes either in optimal conditions or in plants facing environmental cues, including heavy metal (HM) stress. Nowadays, a huge body of evidence has unveiled that SA plays a pivotal role as plant growth regulator and influences intra- and inter-plant communication attributable to its methyl ester form, methyl salicylate, which is highly volatile. Under stress, including HM stress, SA interacts with other plant hormones (e.g., auxins, abscisic acid, gibberellin) and promotes the stimulation of antioxidant compounds and enzymes thereby alerting HM-treated plants and helping in counteracting HM stress. The present literature survey reviews recent literature concerning the roles of SA in plants suffering from HM stress with the aim of providing a comprehensive picture about SA and HM, in order to orientate the direction of future research on this topic.

## 1. Introduction

Salicylic acid (SA) (from Latin *Salix*, willow tree), also known as *ortho*-hydroxybenzoic acid, is a phenolic derivative widely distributed in the plant kingdom and is known as a regulator of several physiological and biochemical processes such as thermogenesis, plant signaling or plant defense, and response to biotic and abiotic stress [1,2].

From a chemical point of view, SA belongs to a large group of plant phenolics, and SA can be isolated in plants in both free and conjugated form. In particular, the conjugated form proceeds from the methylation, hydroxylation, and/or glucosylation of the aromatic ring [3,4].

Salicin, one of the natural SA derivatives, was first isolated from the bark of the willow tree (*Salix* sp.) by Johan Büchner in 1828 [5,6]. Successively, it was discovered that almost all the willow trees including *Salix alba*, *S. purpurea, S. fragilis,* and *S. daphnoides* were particularly rich in this natural compound, in which the concentration in plants significantly fluctuates during the different seasons (highest content during spring and summer, lowest content during autumn and winter [7]) reaching values of 3 mg/g of fresh biomass in plants of *S. laponum* [8]. The first scientist who was able to identify this natural compound in species different from Salix sp. was the Italian chemist Raffaele Piria in the late 1838, who obtained SA in both flower and buds of the European species *Spiraea ulmaria* successively renamed as *Filipendula ulmaria* (L.) Maxim. The discovery that this molecule was not exclusive to the *Salix* genus has opened the door to the study of its biosynthesis, as well as its biochemical and physiological role in plants and in 1899 the Bayer Company formulated a new drug known today as aspirin [9].

**Figure 1:**
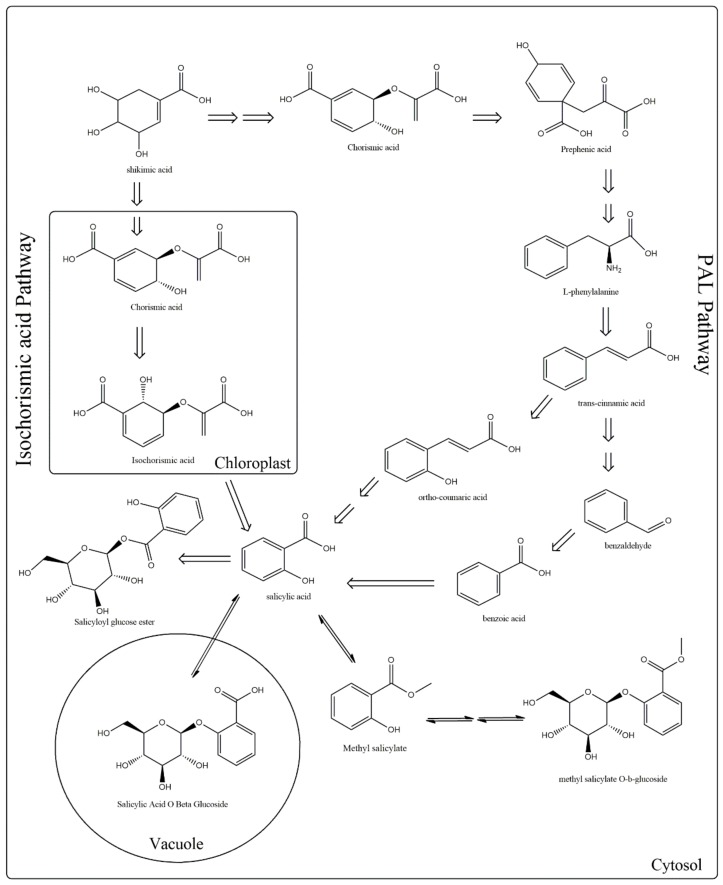
Metabolic pathways involved in the biosynthesis of salicylic acid (SA). Plants use two pathways for SA production, the phenylalanine ammonia-lyase (PAL) (which is divided into two sub-pathways, benzoic acid, and o-coumaric acid) and the isochorismate. In both routes, shikimate serves as a precursor.

Concerning the biosynthesis of SA, it is known to be produced through the shikimate pathway by two metabolic routes (Figure 1). In the first discovered route, also known as phenylalanine route, occuring in the cytoplasm of the cell, the enzyme phenylalanine ammonia lyase (PAL) converts phenylalanine (Phe) to *trans*-cinnamic acid (t-CA), which gets oxidized to benzoic acid (BA). Subsequently, the enzyme benzoic-acid-2-hydroxylase (BA2H) catalyzes the hydroxylation of BA aromatic ring and leads to SA formation. The enzymatic conversion of BA into SA by BA2H requires the presence of hydrogen peroxide (H_2_O_2_) [10,11,12].

The first evidences for the first route were given by Ellis and Amrchein [13], who observed that feeding *Gaultheria procumbens* plants with labeled 14C-benzoic acid or 14C-cinnamic acid resulted in the production of labeled SA. Successively, Yalpani et al. [14] and Silverman et al. [15], working on rice and tobacco, proposed that the side chain of *trans*-cinnamic acid is decarboxylated to generate BA. Then, BA is hydroxylated at the C2 position forming SA. Anyway, recent results indicated that benzoyl glucose, a conjugated form of BA, is more likely to be the direct precursor of SA [12,14].

The second route is called isochorismate (IC) pathway and occurs in the chloroplast [16,17,18]. In plants, chorismate is transformed to isochorismate and then to SA, a reaction which is catalyzed by two enzymes isochorismate synthase (ICS) and isochorismate pyruvate lyase (IPL). Recent studies carried on *Arabidopsis thaliana* demonstrated that the ~90% of defense-related SA is produced from isochorismate generated by the plastid-localized isochorismate synthase1, whereas ~10% is derived from the cytosolic PAL pathway [1,17].

From the physiological point of view, it is known that SA plays a pivotal role in the regulation of plant growth, development, in defense from biotic and abiotic stress, and in plant immune responses [4,19,20,21,22,23].

For several years, SA was believed to be just one of the several phenolic compounds synthetized by plants with relatively low importance [5,16]. In 1974, after more than a hundred of years from its discovery, it was provided the first evidence that SA could play a role as plant hormone, when Clealand and Ajami [24] observed that SA was a mobile signaling molecule localized in the phloem inducing flowering in different plant species.

However, the final evidence that SA was a plant hormone was only provided several years later by Raskin et al. [25], who described its role during the thermogenesis in *Sauromatum guttatum*.

From that moment, an exponential increase of manuscripts focused on SA (acting alone or in concert with other plant hormones) as a plant growth regulator, signaling molecule, as well as plant elicitor protecting plants from biotic and abiotic stress, was observed [22,23,26,27,28,29,30,31].

Recently, it has also been demonstrated that SA could play a pivotal role in protecting plants from environmental stress, including heavy metals (HM). In fact, several recent manuscripts reported that SA can alleviate HM toxicity influencing both their uptake and/or accumulation in plant organs [32,33,34,35,36,37,38], as well as scavenging of reactive oxygen species (ROS) and/or decreasing their accumulation and/or enhancing the antioxidant defense system [39,40,41,42], protecting membrane stability and integrity [43], interacting with plant hormones [44], upregulating heme oxygenase [45], and improving the performance of the photosynthetic machinery [42,46,47].

Focusing on these aspects, the present review provides a comprehensive assemblage concerning SA roles in plant defense from HM stress, with the aim to provide a clear view of SA and HM to orientate the direction of future research on this topic.

## 2. HM Stress and Its Impacts on Plants

Metals and metalloids with atomic density more than 6 g cm^−3^ are defined as (HM). Both, essential elements, micronutrients that are required in low concentration (e.g., Cu, Cr, Co, and Zn), and nonessential metals such as Pb, Cd, Hg, are incorporated in this group [48,49]. Increased concentration of both essential and nonessential elements is phytotoxic to flora and fauna [50,51]. Heavy metal contamination has become a serious environmental problem worldwide. The increased industrialization, injudicious population growth, and urbanization releases HM that compromise soil and water and pose harms to living biota due to their biomagnification through the food chain [52]. Natural activities such as eruption of volcano and erosion of rocks have contribute in increasing the release of toxic elements to the environment; however, increased human activities such as mining, painting, and refining have enhanced their concentration in the biosphere [53,54,55].

Soil pollution by HM poses serious concerns to the biotic and abiotic components of the ecosystem [56]. The increased amount of HM in soil leads to greater uptake by plants that can reduce plant growth, biomass, photosynthesis, crop yield, and quality in plant [57]. From a biological point of view, the top soil is the most active zone of soil that accumulates a large amount of toxic metals that poses serious concern to the environment [49,58,59].

The increased level of HM accumulation in plant organs negatively affects the cell metabolism in plants [60]. The different physiological activities in plants such as protein metabolism, photosynthesis, respiration, and morphogenesis are naturally affected by a high concentration of toxic compounds, such as HM [53,54,61,62]. For instance, Rascio et al. [63] documented a decreased root growth and altered morphogenesis in rice seedlings upon treatment with Cd. Many plant species such as *Brassica napus*, *Helianthus annuus*, *Thalaspi caerulescens*, *Vigna radiata* showed inhibition in photosynthesis in response to Cd treatment [64,65,66,67,68]. Recently, Tandon and Srivastava [69] investigated the Pb effect on the morphology and metabolism of *Sesamum indicum* and found that the increasing concentration of metal affected the growth of the plant. Further, the plant showed severe symptoms of chlorosis, necrosis and reduced chlorophyll, and protein content at higher doses of Pb [69].

The major outcome of metal toxicity is the peaked production of ROS due to impairment of photosynthetic process by HM [70]. ROS such as hydroxyl, superoxide, and hydrogen peroxide are produced as by-product during electron transport in photosynthesis and respiration pathways [71]. Under physiological conditions, ROS play a multitude of signaling roles in plants, as well as in other organisms and they take part in a finely-tuned and well-orchestrated regulatory network [72,73]. ROS are indeed integrated into a complex regulatory system in plants which encompasses ROS, plant hormones (e.g., ethylene (ET) and abscisic acid (ABA)), signaling molecules (e.g., salicylic acid (SA) and jasmonic acid (JA)), and secondary messengers (e.g., Ca^2+^) [74,75]. However, when ROS production exceeds the physiological levels, their accumulation can lead to oxidative stress in the cells, that cause lipids peroxidation, macromolecular degradation, membrane disruption, DNA breakage, and ion leakage in plants [70,74,75]. For instance, Kaur et al. [76] explored Pb-induced ultrastructural changes in roots of wheat and concluded that Pb inhibited root growth, caused ROS generation, and disrupted mitochondrial and nuclear integrity in the tested plant.

The enhanced generation of ROS in the plant cell is controlled by a complex network of antioxidant machinery that maintains ROS homeostasis in the cell [77]. Plants have a finely-tuned and well-orchestrated defense system that includes enzymatic antioxidants such as catalase (CAT), superoxide dismutase (SOD), ascorbate peroxidase (APX), glutathione peroxidase (GPX) and glutathione reductase (GR), and nonenzymatic antioxidants such as ascorbic acid, glutathione, alkaloids, phenol compounds, and α-tocopherol for scavenging excessive ROS [49,61]. Moreover, phytohormones such as auxins, gibberellins, cytokinins, abscisic acid, ethylene, brassinosteroids, jasmonic acid, and SA take part in the defensive mechanism of plants against HM stress.

## 3. Physiological Roles of SA in Plants Under HM Stress

Concerning the physiological role in plants, SA is known to play a pivotal role in regulating plant morphology, development, flowering, and stomatal closure [78,79]. SA also affects seedling germination, cell growth, and nodulation in legumes [80]. Khan et al. [81] reported increased leaf area and dry weight production in corn and soybean in response to SA. Furthermore, Hussein et al. [82] reported pot studies that documented improved growth, leaf number, dry biomass, and stem diameter in wheat plants when leaves were sprayed with SA. The rate of transpiration and stomatal index of plants increased in response to supplementation of SA [81]. The pigment concentration in wheat seeds significantly enhanced upon exposure to a low concentration (10^−5^ M) of SA. However, foliar application of SA reduced transpiration rate in test plants, *Phaseolus vulgaris* and *Commelina communis* which might be due to the SA-evoked stomatal closure [83,84,85,86,87]. Moreover, SA has been reported to increase the shelf life of cut flowers of rose and defer senescence by controlling water level in rose plants [86].

Plant growth regulators or phytohormones especially, gibberellins, auxin, cytokinins, ethylene, brassinosteroids, and also SA play a key role in providing HM tolerance in plants [83]. SA, a phenolic plant hormone, regulates photosynthesis, respiration, and antioxidant defense mechanism in plants under different abiotic stress such as high temperature, salinity, and HM [78,88,89]. SA pretreatment provides protection from various metals such as Pb, Hg, Cd, in different plants [90,91,92].

Supplementation of SA in combination with plant growth promoting bacteria reduces Cr-induced oxidative damage in maize by enhancing activities of antioxidant and nonantioxidant enzymes [93,94]. Earlier, Song et al. [95] reported SA mediated enhancement in the activities of CAT and SOD enzymes in barley leaves under Zn, Cu, and Mn stress. Further, carbohydrate metabolism in Cr-treated maize plants improved upon exposure to SA [94]. Alleviation of Cd toxicity was reported in mustard plants in response to exogenous treatment of SA [93]. Recently, SA treatment mitigated Cd stress in *Brassica juncea* plants and enhanced growth and photosynthesis in plants. Moreover, supplementation of SA reduced reactive oxygen species levels by strengthening the antioxidant defense system in plants and provides stability to the plant membrane [96]. The exogenous application of SA upregulates the antioxidant system, improves growth and yield, and results in lowering of oxidative damage under Pb stress in *B. campestris* [97].

**Figure 2:**
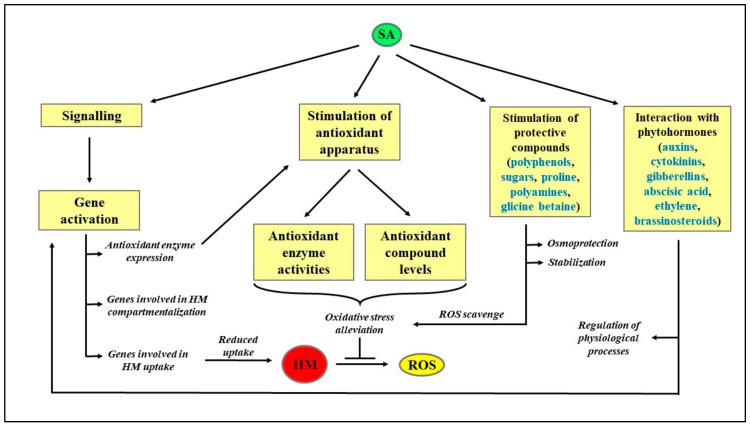
Schematization of the protective role exerted by SA in HM-stressed plants. HM: Heavy metals; ROS: Reactive oxygen species; SA: Salicylic acid.

A schematization of the protective role exerted by SA in HM-stressed plants is reported in Figure 2, whereas a literature survey on the effect of different HM on plant metabolism is reported in Table 1.

### 3.1. Effect of SA to Photosynthesis in Plants Subjected to HM Stress

The different stressful conditions encountered by plants affect multiple physiological and biochemical mechanisms in plants. Among these, photosynthesis is usually one of the most affected mechanisms by HM (see a schematization of the effect of HM on chloroplast in Figure 2). HM accumulated in various organs of plants and affect the synthesis of photosynthetic pigments, including carotenoids and chlorophylls [53,54]. HM also alter the chloroplast membrane structure and affect electron transport, thus impairing light-dependent reactions of photosynthesis [120]. Moreover, it was found that the negative effect of HM on PSI and PSII depends on exposure time and concentrations [121,122]. Experiments performed by Khan et al. [123] indicated that PSII is more sensitive to HM stress compared to PSI, however, at high concentrations the activity of PSI resulted inhibited as well. Photosynthesis inhibition caused by HM is also attributable to the impairment of stomatal conductance and transpiration rate [124].

Plants are equipped with multiple mechanisms to preserve the photosynthetic machinery from HM-promoted damages. SA is a major photosynthesis regulator which influences chlorophyll content, stomatal conductivity, and photosynthesis-related enzyme activities in plants [125]. It enhances photosynthetic efficiency and improves photosynthetic apparatus under HM stress [34]. Exogenous application of SA (500 μM) enhanced chlorophyll concentration, CO_2_ fixation, and activities of phosphoenolpyruvate carboxylase and RuBISCO in *Triticum aestivum* under Cd toxicity [126]. Further, gas exchange parameters and carbonic anhydrase improved in *B. juncea* under Ni [120] and Mn [127] stress after the exposure to 10 μM SA. SA treatment enhanced Chl*a*, Chl*b*, and carotenoid content in barley plants under Pb stress by increasing antioxidant activity in the plants which might be due to blockage of Ca channels that help in translocation of Pb in roots [60]. Recently, Guo et al. [38] studied the role of SA in Cd alleviation and accumulation in tomato plants. The exogenous exposure of SA also increased pigment content and photosynthetic performance in tomato plants [38]. The consistently observed protective role of SA to the photosynthetic apparatus might be due to increased detoxification of ROS species exerted by SA or by the activation of antioxidant apparatus promoted by SA [125].

### 3.2. Regulation Mechanism of ROS and Enzymatic Antioxidants Promoted by SA Acid under HM Stress

The generation of ROS is one the first response in plants under HM stress. ROS production is either directly due to Haber-Weiss reaction or it is indirectly because of interference in the antioxidant defense system or electron transport chain [128]. ROS (H_2_O_2_; hydrogen peroxide, OH^·^; hydroxyl radical, and O_2_^−·^; superoxide radical) are very harmful to plants since they lead to oxidative degeneration of cell membranes and large macromolecules [129]. Plants possess a powerful antioxidant apparatus to counteract oxidative stress, which includes different enzymes (SOD, CAT, APX, GR) and nonenzymatic antioxidants (e.g., glutathione, ascorbic acid, phenolics, carotenoids) that scavenge and detoxify ROS over-production in plants [130].

Lipid peroxidation is the first oxidative injury in plants due to HM stress and SA have been shown to provide stability against HM-induced oxidative damage by increasing antioxidant machinery in plants [125]. Parashar et al. [127] and Zhang et al. [131] observed the reduction in lipid peroxidation, electrolyte leakage, and superoxide ion in Mn- and Cd-treated *B. juncea* and *Cucumis melo* upon addition of SA. Few experiments suggest that SA can promote free radical scavenging of HM-promoted ROS by regulating antioxidant enzymes and expression of some proteins and molecules such as OsWRKY45 as reported in rice by Chao et al. [132] that lowers H_2_O_2_ accumulation in plants. This helps in maintaining the balance between ROS generation and membrane integrity, thereby preventing membrane disruption [133]. Recently, Lu et al. [98] and Gu et al. [99] documented activation of antioxidant enzymes including SOD, APX, and other peroxidases in *Lemna minor* and *Nymphaea tetragona* upon supplementation of SA in plants subjected to Cd stress, which were helpful in conferring Cd tolerance in plants.

### 3.3. Regulation of Osmolytes and Polyphenols by SA under HM Stress

Plants have evolved various mechanisms to counteract HM-triggered ROS production. Different antioxidant metabolites such as proline, glycine betaine, polyamines, sugars, and polyphenols are all involved in maintaining the ROS balance in plants under stressful conditions, including excess of HM. Below, the intimal connections between SA and other antioxidant compounds are described with the attempt to provide a clear and exhaustive picture about the SA-promoted regulation of antioxidant molecules in plants exposed to HM.

#### 3.3.1. Proline

Proline acts as a free radical scavenger, osmo-protectant, and stabilizer of cellular structures [130,134]. The synthesis of proline occurs from glutamate, which is converted to glutamate-semialdehyde, and then spontaneously to pyrroline-5-carboxylate (P5C) with the help of P5C synthase enzyme. Later, the enzyme P5C reductase aids in the reduction of P5C to proline. The stimulation of proline levels under HM stress was observed, for example, in *Olea europaea* [135] and *Phoenix dactylifera* [136]. However, this is not clear whether the accumulation was attributable to enhanced production of enzymes responsible for proline synthesis, the decrease in enzymes related to its oxidation or both. SA is involved in enhancing proline level under HM toxicity [96]. Parashar et al. [127] reported that SA ameliorated the Mn stress through enhanced accumulation of proline in *Brassica juncea* which might be due to the increased activity of enzymes responsible for proline synthesis [137]. Enhanced proline content also maintains water balance in plants to contrast stressful conditions leading to osmotic stress [138] a condition which can occur when plants reduce the stomatal conductance in order to reduce HM uptake. Further, Chen and Dickman [139] proposed that proline is a powerful ROS scavenger and a pivotal component of protein pathway in plants, besides serving as an osmoprotectant [140]. Zanganeh et al. [141] observed however that SA pre-treatment decreased proline accumulation in *Zea mays* under Pb stress that was supported by the findings of Mostafa et al. [142] in rice plants. Therefore, the pattern of proline (activation/decrement) can be species- or metal-specific and also dependent on the dose of HM experienced by the plant species.

#### 3.3.2. Glycine Betaine

Glycine betaine (GB) is a quaternary level ammonium compound found in higher plants under stress conditions and it acts as osmoprotectant or compatible solutes in plants [143], in which it accumulates at cytosolic level. GB is involved in providing protection against drought, salinity [93], drought [143], and HM stress, as well [144]. Exogenous application of GB is very effective in providing tolerance from HM stress [94,145]. The role of SA in regulating the accumulation of GB in plants under metal stress is still unknown. However, few studies reported that exogenous treatment of GB together with SA can help in alleviating HM toxicity [145]. Recently, Aldesuquy et al. [146] opined that GB and SA regulates osmotic pressure and concentration of osmolytes in plants that maintain osmotic balance and helps in ameliorating the adverse effect of drought stress in wheat, thereby suggesting a possible cooperation. It was also reported that the SA induced the rise in GB level which helped the growth of *Rauwolfia serpentina* plants grown under Na excess [147].

#### 3.3.3. Sugars

The term sugars, collectively used for disaccharides (sucrose, trehalose) and fructans, are water-soluble carbohydrates involved in plant stress tolerance. Sucrose, an important product of photosynthesis, is required for growth, development, storage, and signaling in plants [148,149]. Carbohydrates are building blocks of plants that provide energy and act as a signaling molecule during transcriptional, post-transcriptional processes [150]. Accumulation of soluble sugars has been observed in plants under stressful conditions which indicate their role as osmoprotectant and in maintaining cellular balance in plants [151,152]. The exogenous addition of SA enhanced the amount of polysaccharides and sugars in plants and helped in improving their growth [153]. El-tayeb et al. [154] observed that SA provided Cu tolerance in *Helianthus annuus*. The authors reported an increasing level of soluble sugars in plants treated with SA that protects the photosynthetic pigments from Cu toxicity [154]. Similarly, 0.01 M SA enhanced growth and sugar accumulation in tomato plants and provided stress avoidance and tolerance against Na toxicity [155].

#### 3.3.4. Polyamines

Polyamines (PAs) are water-soluble molecules that play an important role in regulating morphological, developmental, and stress responses in plants [156]. PA have the potential to scavenge HM-triggered ROS [157] and regulate plant defense response to HM toxicity [156,158]. Under stressful conditions, PA operate as signaling compounds and control ion homeostasis and ion transportation in plants, thus actively participating in stress tolerance [159,160]. Many reports suggest that SA treatment influence PA content in plants [131,161]. Recently, Tajti et al. [162] studied the role of putrescine and spermidine on wheat under Cd stress and also reported increased levels of SA in those plants; however, the exact mechanism involved in SA-mediated HM stress tolerance and the relationship between PA and SA in plants are still unknown.

#### 3.3.5. Polyphenols

Phenolics are one of the largest groups of secondary metabolites which include a plethora of compounds with simple aromatic rings to very complex molecules, such as tannins and lignans. They originate from phenylalanine by the activity of PAL. Many reports have demonstrated that enhanced production of phenolic compounds under HM stress can protect from oxidative damage [163,164]. The accumulation of phenolics is principally driven by increased expression of enzymes responsible for phenylpropanoid biosynthesis such as phenylalanine ammonia-lyase, chalcone synthase, shikimate dehydrogenase, cinnamyl alcohol dehydrogenase, and polyphenol oxidase [165,166]. Many studies have documented the role of phytohormones in enhancing the level of some classes of polyphenols, such as anthocyanins [167,168]. Dong et al. [169] reported increased concentrations of phenolics, such as caffeic acid due to exogenous treatment by SA. Similarly, peaked activity of PAL was observed in *Matricaria chamomilla* plants under Ni and Cd stress with the application of SA [170].

### 3.4. Regulation of Cell Signaling by SA under HM Stress

The HM stress tolerance induced by SA is supportive for its role in stress signaling. The mechanism of tolerance not only depends on the concentration and mode of application of SA but also on the overall status of plants [171]. Abiotic stress not only affects growth and development of plants, but also regulates DNA replication machinery. SA application upregulates the topoisomerase gene and chloroplast elongation factor that help in plant adaptation under stressful conditions [172,173]. Moreover, SA is known to induce expression of *TLC1*, a long terminal repeated retrotransposon family in vivo [171]. This family is transcriptionally activated during stressful conditions and its expression by SA suggests their role in SA-mediated signaling pathways [171]. Another mechanism adopted by SA in regulating HM stress plant response is the increased activity of enzymes involved in AsA-GSH pathway [174]. Both AsA and GSH are active redox compounds that maintain cellular redox balance in plants [175]. SA supplementation also increased SOD and POD level in Cannabis sativa and improved Cd-tolerance [34] which might be related to increased concentration of Ca^2+^ (a second messenger) and H_2_O_2_, that eventually promote the activity of antioxidant enzymes which reduce cellular ROS level in plants [176,177].

### 3.5. Crosstalk of SA with Other Plant Growth Regulators

SA regulates different plant responses both under optimal and stressful conditions through the crosstalk with other plant growth regulators or plant hormones [81,178]. The interaction of SA with other hormones such as auxin [179], cytokinin [180], gibberellins [181], abscisic acid [182], ethylene [178], and brassinosteroids [87] has been studied under optimum and stressful environments. The possible outcome of interaction of SA with hormones can be either synergistic or antagonistic under stressful conditions. Recently, Tamás et al. [44] studied the SA regulated alleviation of Cd-stress by restriction of Cd-induced auxin-mediated ROS production in barley roots. The authors suggest that SA treatment reversed indole-3-acetic acid (IAA)-induced stress responses in plants suggesting a role of SA in IAA signaling pathway. Similarly, Agtuca et al. [183] reported an opposite role of IAA and SA in roots of maize. The exogenous application of IAA enhanced lateral growth by depriving primary root growth, while SA increased total root biomass [183].

Exposure to various environmental stresses, such as HM, can enhance ethylene production and induce oxidative stress in plants [175]. The increased ethylene production is due to peaked expression of ethylene-related biosynthetic genes or expression of ethylene-responsive genes [184]. Exogenous SA was reported to mitigate Cd stress in wheat [174] by increasing GSH content that resulted in metal detoxification and scavenging ROS induced by HM-triggered ethylene production. SA supplementation promoted increased ABA level in wheat seedlings under Cd stress that was attributed to a de novo ABA biosynthesis [185]. Further, endogenous ABA controlled SA-mediated alteration of the concentration of dehydrin proteins under HM stress that demonstrate protective mechanism of SA in wheat plants [185].

Under abiotic stress conditions, crosstalk between SA and jasmonates play a crucial role in regulation of plant growth [186]. Generally, SA and jasmonic acid (JA) signaling pathways work in an antagonistic manner [187]. The Mitogen-activated protein kinase (MAPK) signaling pathway mediates the antagonistic action between SA and JA cell signaling [188]. However, nonantagonistic interaction between SA and JA are also reported, but an exact mechanism is still unclear and it needs further studies [186]. For example, in maize plants Cu stress induced the biosynthesis of SA, which further induced JA priming and JA induced volatile organic compounds [189,190].

## 4. Conclusions

Heavy metal stress has been accepted as one of the major threats for plants growing in contaminated areas. In order to deal with the harmful effects of heavy metals, plants have developed several molecular, metabolic, and physiological processes which allow them to avoid stressful factors or cope with them.

Several researches highlighted that SA, when used at low doses, plays a pivotal role in both alleviating and reducing heavy metal stress in plants. An increase in the endogenous level, as well as exogenous application of this plant hormone has been demonstrated to be helpful for plants either in optimal or in stress conditions. In fact, this ubiquitous plant hormone is involved in the regulation of several metabolic processes in plants, regulating the ex novo biosynthesis of secondary metabolites and osmoprotectants involved in the protection from oxidative stress, thereby increasing the activity of ROS scavenger enzymes and/or acting as antioxidants. However, at high concentrations SA can also act as a negative plant growth regulator [171,191,192].

The scientific literature cited in the present review highlights the important role played by SA in protecting plants from heavy metal stress. However, most of the researches available on this topic are mainly focused on the role played by this molecule after an exogenous application, while very few researches, because of the complexity of the cascade effects generated, have unveiled the defense mechanisms triggered by its endogenous stimulation in response to heavy metals. Therefore, there are still several questions which need further investigation. For example, it would be extremely interesting to disentangle the complexity of SA signaling in response to heavy metals, as well as to unveil if exogenous application of SA might directly or indirectly enhance endogenous SA levels. In the meantime, more genomic, transcriptomic, proteomic, and metabolomics studies are necessary to detect SA responsive genes, proteins, and metabolites altered by heavy metal stress. In addition, it is necessary that a molecular dissection deeply understands the crosstalk between SA with other phytohormones and/or metabolites and the feedback processes involved in controlling the endogenous levels of SA in response to heavy metal stress.

## Figures and Tables

**Table 1:** Salicylic acid (SA) effect on different heavy metals (HM) stressed plants.

HM	Species	Effects of SA in plant metabolism	References
**Cd**	*Lemna minor* L.	Induced a reduction of Cd uptake, the maintenance of ionic homeostasis, improvement of PAL activity, activation of ROS scavenger and of the heat shock proteins.	[98]
	*Oryza sativa* L.	SA in association with NO reduced Cd uptake and accumulation, as well as ROS accumulation and malondialdehyde production through the maintenance of ascorbate and glutathione levels, and redox status. Improved the activities of antioxidant enzymes such as superoxide dismutase (SOD), catalase (CAT), glutathione S-transferase, and mono dehydroascorbate reductase.	[41]
	*Brassica juncea* L. Czern	Stimulating the stomatal activity and pore size, alleviated the inhibitory effect of Cd on photosynthesis. The Cd-generated oxidative burst was reduced via enhanced antioxidant activity (CAT and SOD) promoted by SA.	[96]
	*Nymphaea tetragona*	SA pretreatment decreased Cd concentration and increased the contents of glutathione, nonprotein thiol and phytochelatins.	[99]
	*Solanum tuberosum*	Cd stress increased endogenous SA level, relative water content, chlorophyll, and proline. Reduced lipid peroxidation, H_2_O_2_ and O_2_^-^. SA stimulated enzymatic antioxidants.	[100]
	*Triticum aestivum L.*	Induced a transient upregulation of protein kinases (SIPK).	[101]
	*Mentha piperita*	Improved photosynthesis by enhancing activity of RuBisCo and carbonic anhydrase. Reduced the oxidative stress by mitigating the production of free radicals by the maintenance of reduced glutathione pool and free radical scavenging enzymes. Furthermore, restored essential oils production previously affected by Cd.	[102]
**Pb**	*Brassica juncea* L. Czern	Co-application of 24-epibrassinolide and SA mitigates the negative effects of Pb, by lowering Pb metal uptake and enhancing the heavy metal tolerance index, antioxidative capacities, organic acid levels, phenolic content, water content, and relative water content.	[37]
	*Zea mays* L.	Improved nitrate reductase activity, glutathione content, and regulated the amino acids metabolism.	[103]
	*Triticum aestivum* L.	Suppressed chlorophyll degradation, electrolyte leakage, and malondialdehyde accumulation. Furthermore, enhanced the production of total soluble carbohydrates, proline, and the activities of SOD, CAT, and peroxidases.	[104]
	*Brassica campestris* L.	Improved plant growth and yield upregulating, in the antioxidant defense system, both enzymatic and nonenzymatic components.	[93]
	*Zea mays* L.	In combination with sodium hydrosulfide reduced arginine, proline, and methionine accumulation and increased nitric oxide and glycine betaine content. Moreover, it regulated the expression of *ZmSAMD* and *ZmACS6* genes (genes involved in methionine metabolism).	[105]
**As**	*Trigonella foenum-graecum* L.	Enhanced root growth and increased protein content, free amino acids, and soluble sugars in both cotyledons and radicles. Moreover, it enhanced the activity of hydrolytic enzymes (α- and β-amylase).	[106]
	*Artemisia annua* L.	Increased endogenous SA level, reduced H_2_O_2_ and O_2_^−^ generation, as well as lipid peroxidation. Reverted biomass and chlorophyll content. Increased artemisinin, and dihydroartemisinic acid level. Upregulated the expression of four key artemisinin biosynthetic pathway genes (*CYP71AV1, ALDH1, ADS,* and *DBR2*).	[107]
	*Artemisia annua* L.	Upregulated proteins related to energy metabolism, photosynthesis, secondary metabolism, transcriptional regulators, transport proteins, and proteins related to lipid metabolism.	[108]
	*Helianthus annuus* L.	Alleviated the negative effect of As on growth and decreased oxidative injuries through the increasing of the enzymatic activity of ROS scavengers such as CAT, ascorbate peroxidase (APX), and glutathione peroxidase, whereas the activity of SOD and guaiacol peroxidase activities was reduced.	[109]
	*Oryza sativa* L.	As enhanced endogenous level of SA and NO level through the enhancement of nitrate reductase activity.	[110]
**Cr**	*Sorghum bicolor* L.	Increased both APX and hydrogen peroxide content and decreased the peroxidase activity and ascorbic acid content.	[111]
	*Brassica napus* L.	Increased dry biomass, enhanced plant growth, and strengthened the reactive oxygen scavenging system by improving the activity in Cr-damaged organelles.	[112]
	*Oryza sativa* L.	Reduced the concentration and translocation of Cr in shoots but not in roots, suggesting a detoxification strategy based on Cr sequestration in roots. Increased growth parameters, membrane stability, and protein content.	[113]
**Ni**	*Brassica juncea* L. Czern. & Coss.	Restored growth and photosynthesis increasing the activities of enzymes associated with antioxidant systems, especially the glyoxalase system and the ascorbate–glutathione cycle (AsA–GSH) cycle. It had an additive effect on the activities of the ascorbate and glutathione pools, and the AsA–GSH enzymes and restored the content of mineral nutrient.	[114]
	*Eleusine coracana* L.	Inhibited Ni transport from roots to shoots, increased chlorophyll content, and the photosynthetic rate, increased the uptake of mineral content, reduced H_2_O_2_ and proline content, and enhanced the activity of antioxidant enzymes (SOD, CAT, APX).	[115]
	*Melissa officinalis* L.	Decreased Ni transport to the shoots, increased carotenoid content, induced a significant decrease in electrolyte leakage in stressed plants.	[116]
	*Alyssum inflatum* Náyr.	Mitigated Ni oxidative effects by reducing H_2_O_2_ concentration. Reversed the detrimental effects of Ni on carotenoid content and reduced the proline content.	[117]
**Co**	*Triticum aestivum* L.	Decreased the accumulation of H_2_O_2_ and MDA and improved the activity of antioxidant enzymes.	[40]
**Cu**	*Gossypium barbadense* L.	Limited Cu translocation and improved the activities of antioxidant enzymes.	[118]
	*Zea mays* L.	Lowered Cu and H_2_O_2_ accumulation in roots. Induced a reduction of MnSODII activity accompanied by a decrease in H_2_O_2_ concentration.	[119]
	*Zea mays* L.	Increased the biomass, root and shoot length, number and leaves area.	[119]
